# Coverage and diagnostic yield of Whole Exome Sequencing for the Evaluation of Cases with Dilated and Hypertrophic Cardiomyopathy

**DOI:** 10.1038/s41598-018-29263-3

**Published:** 2018-07-18

**Authors:** Timothy Shin Heng Mak, Yee-Ki Lee, Clara S. Tang, JoJo S. H. Hai, Xinru Ran, Pak-Chung Sham, Hung-Fat Tse

**Affiliations:** 1 Centre for Genomic Sciences, Li Ka Shing Faculty of Medicine, The University of Hong Kong, Hong Kong, China; 2 Department of Medicine, the University of Hong Kong, Hong Kong, China; 3 Department of Surgery, the University of Hong Kong, Hong Kong, China; 4 The University of Hong Kong – Karolinska Institutet Collaboration in Regenerative Medicine, Hong Kong, China; 5 Department of Psychiatry, the University of Hong Kong, Hong Kong, China; 6 State Key Laboratory of Brain and Cognitive Sciences, The University of Hong Kong, Hong Kong, China; 7 Department of Medicine, Shenzhen Hong Kong University Hospital, Shenzhen, China; 8 Hong Kong-Guangdong Joint Laboratory on Stem Cell and Regenerative Medicine, the University of Hong Kong, Hong Kong, China; 9 Shenzhen Institutes of Research and Innovation, the University of Hong Kong, Hong Kong SAR, China

## Abstract

Targeted next generation sequencing of gene panels has become a popular tool for the genetic diagnosis of hypertrophic (HCM) and dilated cardiomyopathy (DCM). However, it is uncertain whether the use of Whole Exome Sequencing (WES) represents a more effective approach for diagnosis of cases with HCM and DCM. In this study, we performed indirect comparisons of the coverage and diagnostic yield of WES on genes and variants related to HCM and DCM versus 4 different commercial gene panels using 40 HCM and DCM patients, assuming perfect coverage in those panels. We identified 6 pathogenic or likely pathogenic among 14 HCM patients (diagnostic yield 43%). 3 pathogenic or likely pathogenic were found among the 26 DCM patients (diagnostic yield 12%). The coverage was similar to that of four existing commercial gene panels due to the clustering of mutation within MYH7, MYBPC3, TPM1, TNT2, and TTN. Moreover, the coverage of WES appeared inadequate for TNNI3 and PLN. We conclude that most of the pathogenic variants for HCM and DCM can be found within a small number of genes which were covered by all the commercial gene panels, and the application of WES did not increase diagnostic yield.

## Introduction

Dilated cardiomyopathy (DCM) and hypertrophic cardiomyopathy (HCM) are important causes of heart failure^1^ and sudden cardiac deaths^2^. It has been estimated that HCM and DCM affect at least 1/500 and 1/2500 persons, respectively^3,4^. Clinically, DCM is characterized by the dilatation and dysfunction of the left ventricle (LV), while HCM is characterized by the hypertrophy of LV. Both HCM and DCM can be caused by genetic or non-genetic factors, and there are significant overlaps in their clinical phenotypes and etiologies^5^. In particular, mutations in several genes have been reported to cause both DCM and HCM. In the past, genetic testing for cardiomyopathy was usually done by targeted Sanger sequencing of a small number of genes. However, the increased availability of high-throughput genotyping and next generation sequencing (NGS) means that a much larger number of genes can be interrogated at the same time and thus potentially increase the yield in genetic diagnosis^3,6,7^.

NGS can be applied in three ways: targeted sequencing for a number of genes, whole-exome sequencing (WES), and whole-genome sequencing (WGS)^8^. The advantage of targeted sequencing is that the region of sequencing can be highly specific and tailored to the specific application. The region can also be covered at great depth and many samples can be analysed at the same time. For diseases in which only a small number of genes are involved, the cost of targeted sequencing is considerably less than WES and WGS. Indeed, different commercial panels of targeted sequencing have been developed for particular diseases. In contrast, WES attempts to capture and sequence all protein-coding regions in the genome, the capture regions being predesigned through commercial capture kits. The advantage of this approach is that it covers the entire exome and can be used for discovery purposes. However, due to the difficulties in the design of probes, typically the coverage of the exome is not complete. WGS, though more costly than WES, has better performance than WES in terms of the coverage of the genome, even for the exonic regions and also includes the intron regions. However, WES may be sufficient for the genetic diagnosis of cardiomyopathy if the majority of the associated mutations are found in protein coding regions.

More recently, Cirino *et al*.^9^ compared the use of WGS and target sequencing in genetic tests of HCM. They found that WGS was able to identify most of the pathogenic or likely pathogenic variants identified using a targeted HCM panel, and also a number not included in the gene panel. However, one variant from the gene panel was missed due to low coverage. For diseases other than cardiomyopathies, a number of studies recently found that targeted sequencing provided a better coverage than WES for genetic diagnosis^10,11^. Here, we add to this literature by comparing between WES and targeted sequencing used by 4 commercial panels for genetic diagnosis of HCM and DCM.

## Methods

### Study Population

We recruited consecutive Chinese patients with well-characterized phenotypes of DCM or HCM who were followed up in our Cardiac Arrhythmia and Device outpatient clinic at Queen Mary Hospital, Hong Kong between January 2013 and December 2015. The clinical diagnosis of DCM and HCM were made based on current guidelines^12,13^ and verified by two independent cardiologists (JJH and HFT). All patients meeting the diagnositic criteria for DCM and HCM and agreed to genetic testing were recruited. Blood samples were collected for DNA extraction upon recruitment from all patients. This study was approved by the Institutional Review Board of the University of Hong Kong and the Hospital Authority Hong Kong West Cluster, and written informed consent was obtained from each patient.

### Whole Exome Sequencing

All of the libraries were prepared based on the protocols of KAPA Hyper Prep Kit (KR0961-V1.14). Exome capture was prepared based on the protocols of Roche SeqCap EZ Library SR User’s Guide version 4.1. Before hybridization, 6 libraries were normalized and combined with different indices into a single pool prior to enrichment. The pooled DNA libraries were mixed with capture probes of targeted regions using the SeqCap EZ Exome + UTR capture kit. The hybridization was performed at 47 °C for 64–72 hours to ensure targeted regions bind to the capture probes thoroughly. Streptavidin beads were used to capture probes containing the targeted regions of interest. Four wash steps with different wash buffer and temperature were done to remove non-specific binding from the beads.

The enriched libraries on the beads were then amplified by polymerase chain reaction (PCR). The enriched libraries were validated by Agilent Bioanalyzer, Qubit and qPCR for quality control analysis. The libraries were denatured and diluted to optimal concentration and applied in the cluster generation steps. HiSeq PE Cluster Kit v4 with cbot was used for cluster generation on the flow cell. Illumina HiSeq SBS Kit v4 was used for paired-end 101 bp sequencing on an Illumina HiSeq. 1500 machine. The raw data were then processed according to the Genome Analysis Toolkit (GATK) Best practices recommendations^2^ by an in-house pipeline which included alignment using BWA version 0.7.9a and Base quality score recalibration using GATK (version 2.8). The average coverage of the target region of the capture kit was 45, with 69% over 30x and 92% over 10x.

In calling variants in our sample, we used the HaplotypeCaller algorithm from GATK 2.8 and applied Variant Quality Score recalibration (VQSR)^14^. We then used KGGseq.^15^ to filter variants according to their quality scores and depth of coverage. Variants with genotype quality <20, and depth <10 were filtered out, as were variants that had a homozygous reference call but > 5% alternative alleles, variants with a heterozygous call and less than 25% alternative alleles, and variants with a homozygous alternative call but <75% alternative alleles. We also filtered out variants in the least specific tranche after VQSR (99.9% <Sensitivity <100.0%).

### Assessment of coverage of WES and commercial gene panels on potentially pathogenic variants related to HCM and DCM

**Figure 1:**
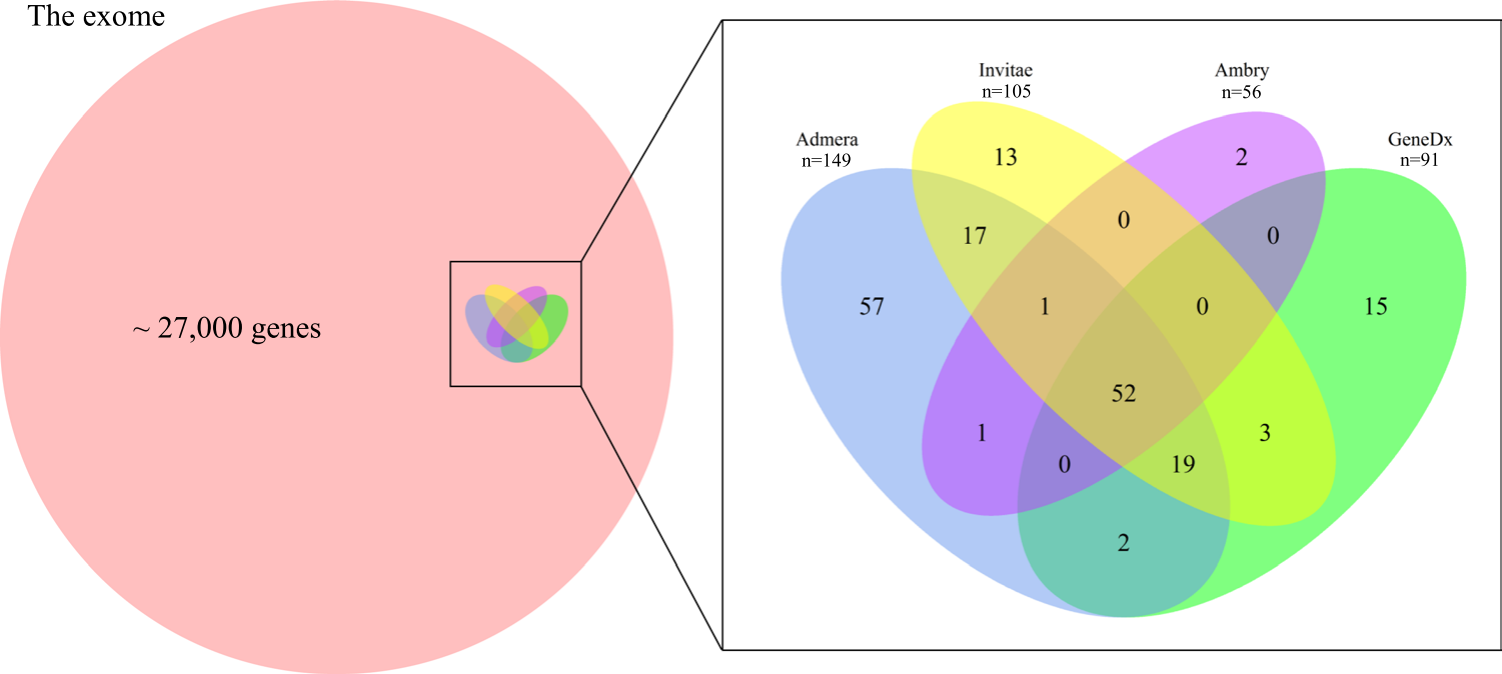
Venn diagram of the number of genes covered by 4 commercial panels in relation to the exome.

We assessed the coverage of the WES with respect to exonic regions defined in the RefGene database^16^, and we compared this coverage with the coverage of cardiomyopathy genes offered by four commercial gene panels from several of the biggest medical genetic testing companies in the United States (GeneDx, Invitae, Ambrygen, Admera – see Web resources for links). As data on the coverage of the various commercial gene panels were not available, we did the comparison under the best-case scenario of assuming the commerical gene panels had perfect (100%) coverage of the genes listed. Where multiple transcripts for a gene existed, the exon regions were taken from the transcript with the longest exon regions. Supplementary Table 1 lists the genes covered by the four panels and Fig. 1 gives the Venn diagram of the genes covered by the four commercial panels. Altogether 182 genes were covered, and less than one third of them were covered by all four panels. The Admera panel was the largest with 149 genes, while Ambry was the smallest with only 56 genes. Some genes were more related to HCM and DCM than others.

In addition to the genes covered by the four commercial panels, we defined a “core” gene list which included only 17 genes associated with HCM or DCM in 6 review papers (Lee *et al*.^17^, McNally *et al*.^7^, Ho *et al*.^18^, Kimura^19^, Xu *et al*.^20^, Hershberger and Siegfried^21^) as well as the OMIM phenotype series PS115200 and PS192600 as listed in Table 1. Moreover, we also assessed the coverage of WES and the commercial gene panels on Clinvar^22^ variants. Variants from the Clinvar^22^ database associated with HCM or DCM with clinical significance “Likely pathogenic” or “pathogenic” were included.

**Table 1:** “Core” genes associated with HCM and DCM as covered by the following reviews (Lee *et al*.^17^, McNally *et al*.^7^, Ho *et al*.^18^, Kimura^19^, Xu *et al*.^20^, Hershberger and Siegfried^21^ as well as the OMIM phenotype series PS115200 and PS192600 (accessed 29/4/2016).

HCM	DCM
*MYH7*, *MYBPC3*, *TNNI3*, TNNT2, *TPM1*, *ACTC1*, *MYL2*, *MYL3*, *CSRP3*	*ACTN2*, *MYBPC3*, *MYH7*, *TNNC1*, *TNNI3*, *TNNT2*, *TPM1*, *TTN*, *PLN*, *ABCC9*, *SCN5A*, *DES*, *LMNA*, *CSRP3*

### Genetic diagnosis of DCM and HCM

WES was applied to our sample to discover pathogenic variants. To identify variants that were pathogenic or likely pathogenic, we looked up the variants’ Minor Allele Frequency (MAF) in the Exome Aggregation Consortium (ExAC)^23^. We used 0.01 as an initial filtering criterion to limit the number of variants considered. If a variant had a review status of 2 or above in Clinvar, and was classified as Pathogenic in causing HCM or DCM using the American College of Medical Genetics and Genomics (ACMG) (2015) guidelines^24^ or by a CLIA (Clinical Laboratory Improvement Amendments) approved lab (such as GeneDx, LMM, and Invitae), it was considered a known pathogenic variant. Other variants were classified by applying the ACMG guidelines manually. Full details of applying the ACMG guidelines are given in Supplementary Table 2. As the application of the ACMG guidelines involved a detailed literature search for each variant, the search was limited to Loss-of-function variants and variants for which a record was found in the Clinvar database with links to HCM or DCM. All data were analyzed with R 3.4.2.

## Results

### Patient characteristics

The study population consisted of 26 patients with DCM and 14 patients with HCM. Their clinical characteristics are summarized in Table 2. While one patient with DCM and 3 patients with HCM had a family history of cardiomyopathy, the others were isolated probands who did not have a living relative with a confirmed diagnosis of cardiomyopathy. The majority of the patients either presented with cardiac arrhythmias or were referred for evaluation for device therapy. As a result, 34/40 (85%) of them were implanted with an implantable cardioverter defibrillator for the prevention of primary (20/34, 58%) or secondary (14/34, 42%) sudden cardiac death.

**Table 2:** Clinical characteristics of 14 HCM and 26 DCM patients in the study.

	HCM (n = 14)	DCM (n = 26)
Mean age (SD)	61.1 (13.7)	57.8 (16.4)
% Female	36%	19%
% Family history of cardiomyopathy	25%	4%
% Family history of sudden cardiac death	17%	4%
% Use of implantable Cardioverter Defibrillator (ICD)	86%	85%
Mean Last Left Ventricular Ejection Fraction (SD)	36.0 (16.4)	56.4 (13.7)
% History of sudden cardiac arrest	0%	35.7%
% Nonsustained ventricular tachycardia	42.3%	71.4%
% Ventricular tachycardia/ventricular fibrillation/ICD shocks	50.0%	35.7%
% Coronary Artery Disease	19.2%	14.3%
% Atrial Fibrillation	34.6%	71.4%

### Coverage of WES

**Figure 2:**
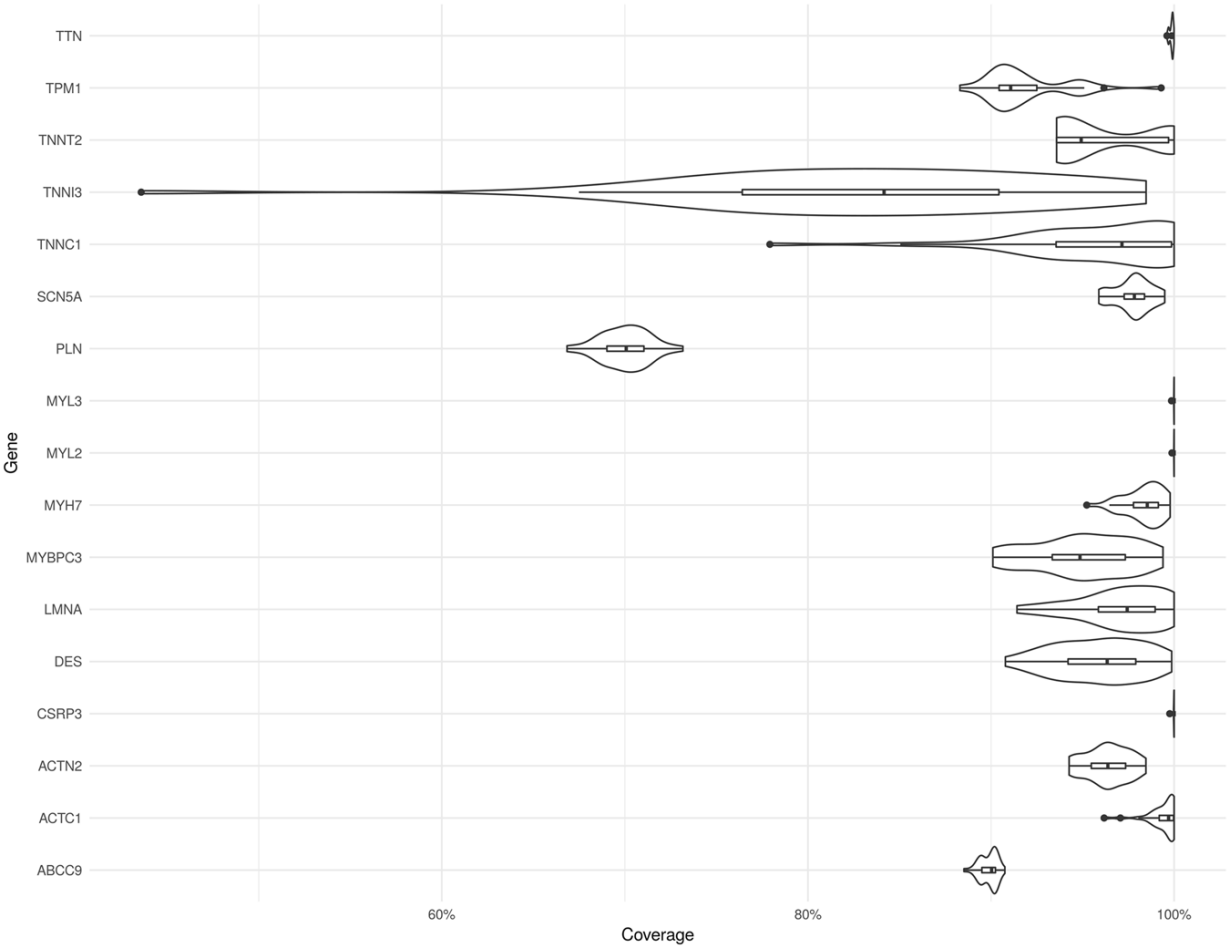
Violin plots of coverage among 17 “core” genes related to HCM and DCM. The plot shows the distribution of coverage among 40 HCM and DCM patients.

Figure 2 summarises the percentage of coverage among 17 “core” genes (Table 1) associated with HCM and DCM among our patients. Coverage is defined by a sequencing depth of at least 10. The coverage of CSRP3, MYL3, MYL2, and TTN was close to 100%. All of the other genes except TNNI3 and PLN had above 90% coverage in most cases. The coverage of TNNI3 and PLN was especially poor. The sample with the lowest coverage of TNNI3 had only 43% coverage. The coverage of PLN was more even among the 40 patients, and was normally distributed around 70%. With reference to the genes covered by the four commercial gene panels, our WES covered 94% to 95% of the exon regions of these genes (Admera: 94%, GeneDx: 95%, Invitae: 94%, Ambry: 94%).

**Figure 3:**
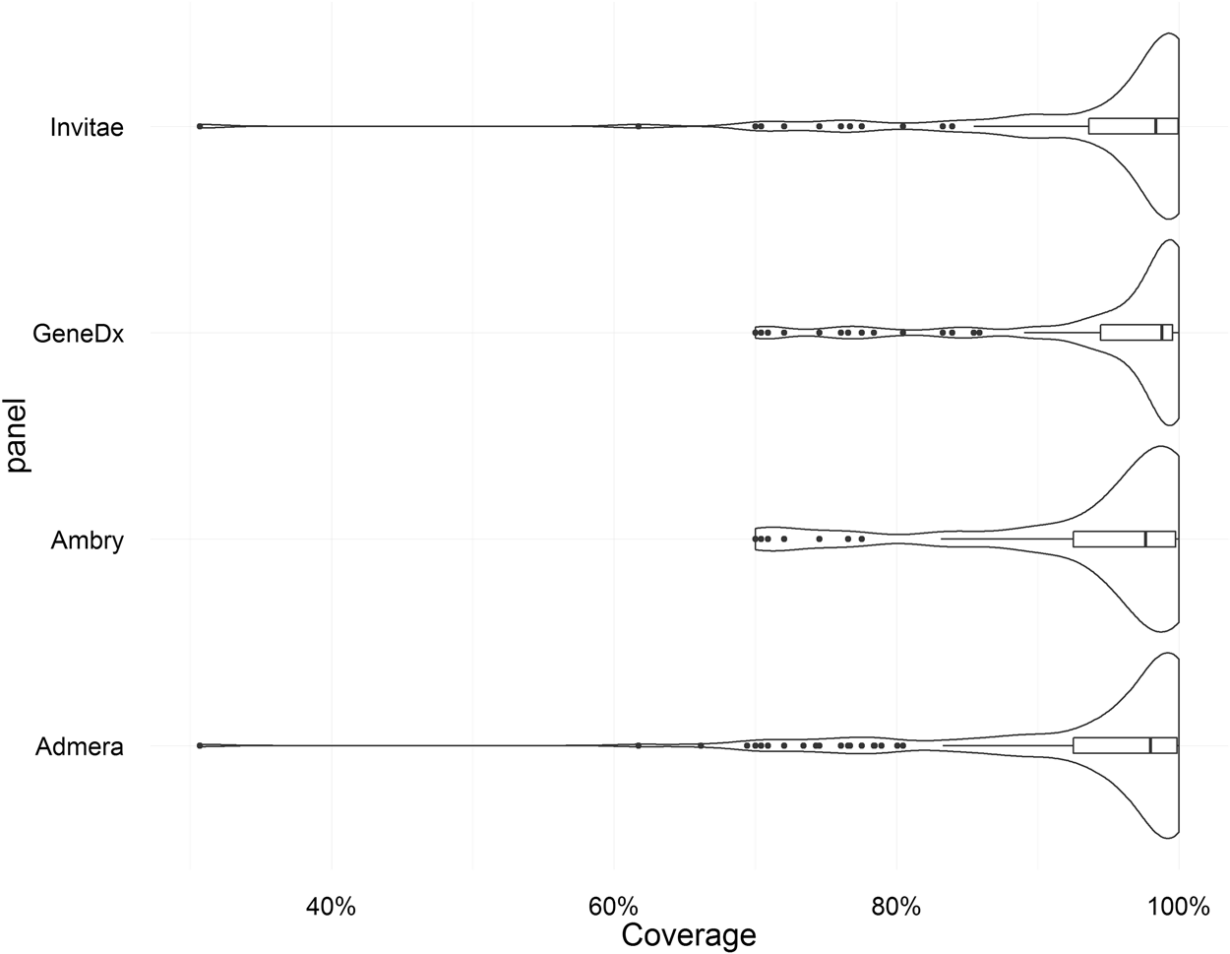
Violin plots of mean WES coverage of the genes covered by four commercial panels. The plot shows the distribution of coverage over the genes.

Figure 3 gives violin plots of the coverage of our WES over the genes covered by these panels. Roughly 80% of the genes had over 90% mean coverage (Admera: 77%, Ambry: 77%, GeneDx: 82%, Invitae: 79%). The gene with the lowest coverage was CTNNA3, with only 30% coverage. This gene was only covered by Admera and Invitae.

### Coverage of WES and gene panels on potentially pathogenic variants for cardiomyopathy

**Figure 4:**
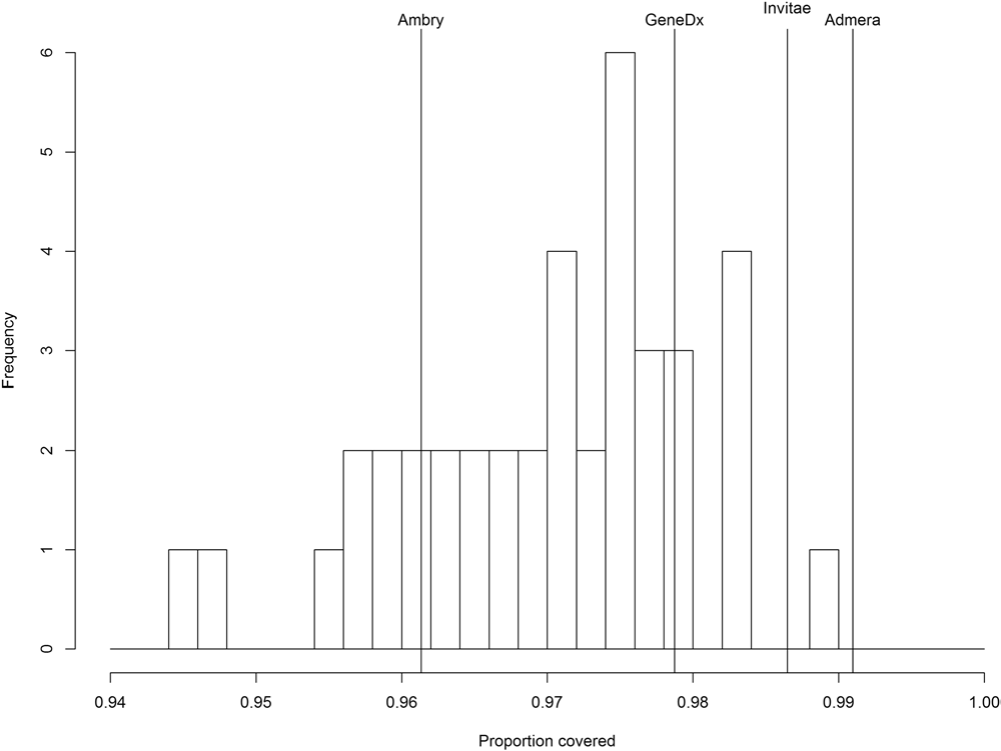
Histogram of WES coverage of 1552 potentially pathogenic cardiomyopathy related variants in Clinvar among 40 HCM and DCM patients. Coverage of commercial gene panels is given as reference. 100% coverage within the genes covered by the panels is assumed.

Figure 4 displays the coverage of the whole exome sequences on 1552 likely pathogenic and pathogenic variants extracted from the Clinvar database. At least 96% of the variants were found in genes covered by the four commercial panels. Coverage of our WES was at a similar level. Across the 40 patients, the mean percentage covered was 97%. All of the samples attained at least 94% coverage. Among the 1552 potentially pathogenic variants, 27 had mean coverage (across patients) less than 10. Among these 27 variants, 8 were in *TNNT2*, one was in *LMNA*, and 2 were in *TNNI3*. All 8 variants for *TNNT2* were located in a small exonic region on chromosome 1 (hg19:201333455–201333497).

### Genetic diagnosis of cardiomyopathy

Using the ACMG guidelines as described in Supplementary Table 2, 2 pathogenic and 4 likely pathogenic variants were identified in 6 of our 14 HCM patients (diagnostic yield 43%). Five of the variants were missense variants, with 4 in the *MYH7* gene, and 1 in *TPM1*. The other was a frameshift mutation in the *MYBPC3* gene. Among our 26 DCM patients, 1 pathogenic and 2 likely pathogenic variants were identified in 3 patients (diagnostic yield 12%). The pathogenic variant was a missense variant in *TNNT2* found in the Clinvar database. The two likely pathogenic variants were a frameshift and a splicing variant found in *TTN*. All these pathogenic and likely pathogenic variants were found in genes that were covered by the 4 commerical gene panels. All except one of the variants were found in patients without a known family history of cardiomyopathy or sudden cardiac death. Full details of the variants identified are tabulated in Table 3.

**Table 3:** List of variants identified in the HCM and DCM patients. MAF: Minor Allele Frequency in ExAC^23^ .

Hg19 position	Gene	rsID	Mutation	Amino acid change	Mutation type	MAF	Pathogenicity criteria
**HCM**							
*Pathogenic*							
11:47372858	MYBPC3		c.A224insG+		frameshift	0	PM1, PM2, PVS1
14:23884860	MYH7	rs193922390	c.5135 G>A	p.R1712Q	missense	8.1e-6	known pathogenic
*Likely Pathogenic*							
14:23894049	MYH7	rs138049878	c.2608 C>T	p.R870C	missense	8.1e-6	PM1, PM5, PP1, PP2
14:23894612	MYH7	rs727503260	c.2302 G>C	p.G768R	missense	0	PS1, PM1, PM2, PP2
14:23899059	MYH7	rs397516088	c.1063 G>A	p.A355T	missense	0	PM1, PM2, PP1, PP2
14:63336299	TPM1	rs199476306	c.188 C>T	p.A63V	missense	0	PS3, PM1, PM2
**DCM**							
*Pathogenic*							
1:201334425	TNNT2	rs121964856	c.260 G>A	p.R87Q	missense	0	known pathogenic
*Likely pathogenic*							
2:179423322	TTN		c.A60242del	p.S20082	frameshift	0	PVS1, PM2
2:179549632	TTN		c.13859 splicing		splicing	0	PVS1, PM2

## Discussion

Targeted NGS has become very popular for the genetic diagnosis of HCM and DCM, since a significant proportion of patients with cardiomyopathy are believed to have genetic causes, and a large number of genes are potentially involved. As the gene list for HCM and DCM continues to grow and the costs of NGS continue to drop, WES may become increasingly viable as an alternative to targeted sequencing. In this study, we used a fairly stringent set of criteria for defining variants as “pathogenic” or “likely pathogenic”, based on the ACMG guidelines^24^. In our sample of 14 HCM patients and 26 DCM patients, WES identified 6 “pathogenic” and “likely pathogenic” variants among the HCM patients and 3 “pathogenic” and “likely pathogenic” among the DCM patients, representing a diagnostic yield of 43% and 12% respectively. Our yield for HCM was slightly higher than Walsh *et al*.^25^ (32%), a recent large American study (Alfares *et al*.^26^, 32%), as well as two Chinese studies (Chiou *et al*.^27^, 34%, Liu *et al*.^28^, 22%). On the other and, our yield for DCM was similar to Walsh *et al*.^25^ (13%), but was less compared to other large studies such as Pugh *et al*.^29^ (37%) and Haas *et al*.^30^ (73%), as well as a Chinese study (Zhao *et al*.^31^, 57%). The large variation in yields for DCM studies is due to the wide variation in definitions of pathogenicity used among the studies as well as the genes that are covered by the targeted sequencing panels.

The “likely pathogenic” and “pathogenic” variants were all found on genes that are closely related to cardiomyopathy. This is partly a result of the fact that our diagnostic criteria is strongly biased towards genes with established pathogenicity. As a result, the use of WES did not increase the diagnostic yield versus the 4 commercial panels. Family studies (e.g. Nomura *et al*.^32^) and functional studies are needed to uncover variants in other genes. If in the future, more variants outside the core genes associated with cardiomyopathy were discovered, WES may prove more useful. Indeed, reanalysis of the same WES data at a later date may provide insight not possible with targeted sequencing data, especially as raw sequencing data are typically not available from the commerical providers.

In this study, we also found that the coverage of WES was not complete even for some of the key genes associated with HCM and DCM. For example, the exon regions of the gene *TNNI3* had only 83% coverage on average and coverage was as low as 43% in one sample. Focusing on 1552 potentially pathogenic variants curated in Clinvar, around 94–99% of all such variants were covered by WES. Nonetheless, a considerable number of variants in *TNNT2*, an important cardiomyopathy gene, were missed. However, as we do not have coverage data for the commercial gene panels, it is possible that this lack of coverage for some genes also applies to targeted sequencing.

### Study limitations

Because of the lack of access to coverage data by the commercial gene panels, we were unable to have a direct comparison between WES and targetted gene sequencing. Moreover, the coverage of WES in the intronic region and for copy number of variations is poor, event though variants related to HCM and DCM have been reported in these regions^33,34^. Whether the use of whole genome sequencing can improve the diagnostic yield in HCM and DCM requires further study. Lastly, our study sample belonged to a group of relatively severe HCM and DCM patients. The diagnostic yield may have been lower if a less severe group was enrolled.

## Conclusions

Our results suggest that WES using the Roche SeqCap EZ capture kit was not complete for all HCM and DCM variants. In our sample of 40 HCM and DCM patients, it did not improve the diagnostic yield for HCM and DCM compared to existing commercial gene panels.

### Web resources

Links to cardiomyopathy panels:

GeneDx – https://www.genedx.com/test-catalog/available-tests/cardiomyopathy-panel.

Invitae – https://www.invitae.com/en/physician/tests/02251/#test-order.

Ambrygen – http://www.ambrygen.com/tests/cmnext.

Admera – http://www.admerahealth.com/cardiogxone/cardiomyopathies-general-panel/.

## Electronic supplementary material

Supplementary Tables
