# Supplementary material for: Coverage and diagnostic yield of Whole Exome Sequencing for the Evaluation of Cases with Dilated and Hypertrophic Cardiomyopathy
Source: Sci Rep. 2018 Jul 18;8:10846. doi: 10.1038/s41598-018-29263-3 (PMC6052112; doi:10.1038/s41598-018-29263-3)
Supplement: Supplementary file 1 — Supplementary Tables [file 41598_2018_29263_MOESM1_ESM.pdf]

Supplementary Tables

Coverage and diagnostic yield of Whole Exome Sequencing for the Evaluation of Cases with Dilated and Hypertrophic Cardiomyopathy

Timothy Shin Heng Mak, Yee-Ki Lee,Clara S Tang, JoJo SH Hai, Xinru Ran, Pak-Chung Sham, Hung-Fat Tse

Supplementary Table S1. Genes covered by three commercial panels for cardiomyopathy

**Company**	**Admera Health**[1]	**GeneDx**[2]	**Invitae**[3]	**Ambry**[4]
**Product**	CardioGxOne (cardiomyopathies general panel)	Cardiomyopathy panel	Cardioyopathies	CMNEXT
**Diseases covered**	HCM, DCM, RCM, ACM, non-compaction cardiomyopathy, RASopathies, storage diseases, and congenital heart diseases	ARVC, DCM, HCM, LVNC, Noonan syndrome	ARVC, DCM, HCM, Fabry disease, LVNC, transthyretin amyloidosis	ARVD, DCM, HCM, LVNC, RCM
**Genes**	AARS2, ABCC9, ACAD9, ACADVL, ACTA1, ACTA2, ACTC1, ACTN2, AGK, AGL, AGPAT2, ALMS1, ANK2, ANKRD1, ATPAF2, BAG3, BRAF, BSCL2, CALR3, CASQ2, CAV3, CBL, COQ2, COX15, COX6B1, CRELD1, CRYAB, CSRP3, CTF1, CTNNA3, DES, DLD, DMD, DNAJC19, DOLK, DSC2, DSG2, DSP, DTNA, ELN, EMD, EYA4, FAH, FHL1, FHL2, FHOD3, FKRP, FKTN, FLNA, FLNC, FOXD4, GAA, GATA4, GATA6, GATAD1, GFM1, GJA1, GJA5, GLA, GLB1, GNPTAB, GUSB, HCN4, HFE, HRAS, JAG1, JPH2, JUP, KCNH2, KCNJ2, KCNJ8, KCNQ1, KLF10,	ABCC9 , ACTC1, ACTN2, ALMS1, ALPK3, ANKRD1, BAG3, BRAF, CAV3, CHRM2, CRYAB , CSRP3, DES, DMD, DOLK, DSC2, DSG2, DSP, DTNA, EMD, FHL1, FKRP, FKTN, GATAD1, GLA, HCN4, HRAS, ILK, JPH2, JUP, KRAS, LAMA4, LAMP2, LDB3 , LMNA, MAP2K1, MAP2K2, MIB1, MTND1, MTND5, MTND6, MTTD, MTTG, MTTH, MTTI, MTTK, MTTL1, MTTL2, MTTM, MTTQ, MTTS1, MTTS2, MURC, MYBPC3, MYH6, MYH7, MYL2, MYL3, MYLK2, MYOZ2, MYPN, NEBL , NEXN, NKX2-5, NRAS, PDLIM3, PKP2,	A2ML1, ABCC9, ACADVL, ACTC1, ACTN2, AGL, ALMS1, ANKRD1, BAG3, BRAF, CACNA1C, CALR3, CAV3, CBL, CHRM2, CPT2, CRYAB, CSRP3, CTF1, CTNNA3, DES, DMD, DNAJC19, DOLK, DSC2, DSG2, DSP, DTNA, ELAC2, EMD, EYA4, FHL1, FHL2, FKRP, FKTN, FLNC, GAA, GATA4, GATA6, GATAD1, GLA, HCN4, HRAS, ILK, JPH2, JUP, KRAS, LAMA4, LAMP2, LDB3, LMNA, LRRC10, MAP2K1, MAP2K2, MTO1, MYBPC3, MYH6, MYH7, MYL2, MYL3, MYLK2, MYOM1, MYOZ2, MYPN, NEBL, NEXN,	ABCC9, ACTC1, ACTN2, ANKRD1, BAG3, CRYAB, CSRP3, DES, DMD, DSC2, DSG2, DSP, EMD, EYA4, FKTN, FXN, GATAD1, GLA, JPH2, JUP, LAMA4, LAMP2, LDB3, LMNA, MYBPC3, MYH6, MYH7, MYL2, MYL3, MYOZ2, MYPN, NEXN, NKX2-5, PKP2, PLN, PRKAG2, PTPN11, RAF1, RBM20, RYR2, SCN5A, TAZ, TBX20, TCAP, TGFB3, TMEM43, TMPO, TNNC1, TNNI3, TNNT2, TPM1, TTN, TTR, TXNRD2, VCL, ZASP

	KRAS, LAMA2, LAMA4, LAMP2, LDB3, LIAS, LMNA, MAP2K1, MAP2K2, MIB1, MLYCD, MRPL3, MRPS22, MTO1, MURC, MYBPC3, MYH11, MYH6, MYH7, MYL2, MYL3, MYLK2, MYOT, MYOZ2, MYPN, NEBL, NEXN, NKX2-5, NOTCH1, NRAS, OBSL1, PDHA1, PDLIM3, PHKA1, PITX2, PKP2, PLN, PMM2, PRDM16, PRKAG2, PSEN1, PSEN2, PTPN11, RAF1, RBM20, RYR2, SCN5A, SGCA, SGCB, SGCD, SHOC2, SLC22A5, SLC25A4, SMAD3, SOS1, SPRED1, SURF1, TAZ, TBX1, TBX20, TBX5, TCAP, TGFB3, TMEM43, TMEM70, TMPO, TNNC1, TNNI3, TNNT2, TPM1, TRIM63, TSFM, TTN, TTR, TXNRD2, VCL (n=149)	PLN, PRDM16, PRKAG2, PTPN11, RAF1, RBM20, RIT1, RYR2, SCN5A, SGCD, SOS1, TAZ, TCAP, TGFB3, TMEM43, TMPO, TNNC1, TNNI3, TNNT2, TPM1, TTN, TTR, TXNRD2, VCL (n=91)	NF1, NKX2-5, NPPA, NRAS, PDLIM3, PKP2, PLEKHM2, PLN, PRDM16, PRKAG2, PTPN11, RAF1, RASA1, RBM20, RIT1, RRAS, RYR2, SCN5A, SDHA, SGCD, SHOC2, SLC22A5, SOS1, SOS2, SPRED1, TAZ, TCAP, TGFB3, TMEM43, TMEM70, TMPO, TNNC1, TNNI3, TNNT2, TPM1, TTN, TTR, TXNRD2, VCL (n=105)	(n=56)

1. http://www.admerahealth.com/cardiogxone/cardiomyopathies-general-panel/
2. https://www.genedx.com/test-catalog/available-tests/cardiomyopathy-panel/
3. https://www.invitae.com/en/physician/tests/02251/#test-order
4. http://www.ambrygen.com/tests/cmne

Supplementary Table S2. Methods for applying the ACMG guidelines

Evidence of pathogenicity	ACMG definition	Our working definition
**Very strong**	PVS1: Null variant (nonsense, frameshift, canonical +/1 1 or 2 splice sites, initiation codon, single or multiexon deletion) in a gene where Truncating is a known mechanism of disease	Truncating variants in genes whose truncating variants are significantly associated with HCM/DCM according to Walsh et al[6] (after correction for multiple testing).
**Strong**	PS1: Same amino acid change as a previously established pathogenic variant regardless of nucleotide change	A previously established pathogenic variant was defined as one which had a Pathogenic call from the ACMG guideline.
	PS2: De novo in a patient with the disease and no family history	based on literature review
	PS3: Well-established in vitro or in vivo functional studies supportive of a damaging effect on the gene or gene product	based on literature review
	PS4: The prevalence of the variant in affected individuals is significantly increased compared with the prevalence in controls	Not applicable.
**Moderate**	PM1: Located in a mutational hot spot and/or critical and well-established functional domain without benign variation	Mutational hotspots for 190 genes were given in the Supplementary material of Maxwell et al[7]
	PM2: Absent from controls in Exome sequencing project, 1000 Genomes Project, or Exome Aggregation Consortium	Absent in the Exac database, the 1000 Genome database, and a cohort of 712 local DDD patients
	PM3: For recessive disorders, detected in trans with a pathogenic variant	Not applicable
	PM4: Protein length changes as a result of in-frame deletions/insertions in a nonrepeat region or stop-loss variants	nonframeshift variants
	PM5: Novel missense change at an amino acid residue where a different missense change determined to be pathogenic has been seen before	Like PS1, a previously established pathogenic variant was defined as one which had a Pathogenic call from the ACMG guideline.
	PM6: Assumed de novo, but without confirmation of paternity and maternity	based on literature review
**Supporting**	PP1: Cosegregation with disease in multiple affected family members in a gene definitively known to cause the disease	If evidence found in Clinvar.

Evidence of pathogenicity	ACMG definition	Our working definition
	PP2: Missense variant in a gene that has a low rate of benign missense variation and in which missense variants are a common mechanism of disease	non-truncating variant found in gene whose RVIS (Residual Variation Intolerance Score) [1] is in the most intolerant 10%, or<br>Truncating variant found in gene whose pLI (probability of Loss-of-function intolerant) [2] score is in the most intolerant 10%.
	PP3: Multiple lines of computational evidence support a deleterious effect on the gene or gene product	Predicted to be pathogenic by Polyphen (HDIV), Polyphen (HVAR)[3], deleterious by SIFT (Sorting Intolerant From Tolerant) [4],and Mutation Taster[5].
	PP4: Patient's phenotype or family history is highly specific for a disease with a single genetic etiology	Not applicable
	PP5: Reputable source recently reports variant as pathogenic, but the evidence is not available to the laboratory to perform an independent evaluation	Not applicable
**Benign (stand-alone)**	BA1: Allele frequency is >5% in Exome sequencing project, 1000 Genome project, or Exome Aggregation consortium	Not applicable
**Benign (Strong)**	BS1: Allele frequency is greater than expected for disorder	MAF > 0.1%
	BS2: Observed in a healthy adult individual with full penetrance expected at an early age	Not applicable. Cardiomyopathy not fully penetrant at early age
	BS3: Well-established in vitro or in vivo function studies show no damaging effect on protein function or splicing	Literature review
	BS4: Lack of segregation in affected members of a family	Literature review
**Benign (Supporting)**	BP1: Missense variant in a gene for which primarily truncating variants are known to cause disease	Missense variants in TTN or SCN5A (according to Walsh et al)
	BP2: Observed in trans with a pathogenic variant for a fully penetrant dominant gene/disorder or observed in cis with a pathogenic variant in any inheritance pattern	Not applicable. Information not available.
	BP3: In-frame deletions/insertions in a repetitive region without a known function	Not applicable. Information not available.
	BP4: Multiple lines of computational evidence suggest no impact on gene or gene product	Predicted to be benign by Polyphen (HDIV), Polyphen (HVAR)[4], deleterious by SIFT[5],and Mutation Taster[3].

Evidence of pathogenicity	ACMG definition	Our working definition
	BP5: Variant found in a case with a an alternate molecular basis for disease	awarded to variants found in patients with variants classified as “Pathogenic”
	BP6: Reputable source recently reports variant as benign	One “Benign” or “Likely benign” classification in Clinvar
	BP7: A synonymous variant for which splicing prediction algorithms predict no impact to the splice consensus sequence nor the creation of a new splice site and the nucleotide is not highly conserved	Not applicable. Synonymous variants are not considered

References:

1. Petrovski, S., Wang, Q., Heinzen, E. L., Allen, A. S. & Goldstein, D. B. Genic Intolerance to Functional Variation and the Interpretation of Personal Genomes. *PLoS Genet.* **9,** (2013).
2. Lek, M. *et al.* Analysis of protein-coding genetic variation in 60,706 humans. *Nature* **536,** 285–291 (2016).
3. Adzhubei, I. A. *et al.* A method and server for predicting damaging missense mutations. *Nat. Methods* **7,** 248–249 (2010).
4. Kumar, P., Henikoff, S. & Ng, P. C. Predicting the effects of coding non-synonymous variants on protein function using the SIFT algorithm. *Nat. Protoc.* **4,** 1073–1081 (2009).
5. Schwarz, J. M., Cooper, D. N., Schuelke, M. & Seelow, D. MutationTaster2: mutation prediction for the deep-sequencing age. *Nat. Methods* **11,** 361–2 (2014).
6. Walsh, R. *et al.* Reassessment of Mendelian gene pathogenicity using 7,855 cardiomyopathy cases and 60,706 reference samples. *Genet. Med.* 41111 (2016). doi:10.1038/gim.2016.90
7. Maxwell, K. N. *et al.* Evaluation of ACMG-Guideline-Based Variant Classification of Cancer Susceptibility and Non-Cancer-Associated Genes in Families Affected by Breast Cancer. *Am. J. Hum. Genet.* **98,** 801–817 (2016).
